# Author Correction: Multipurpose silicon photonics signal processor core

**DOI:** 10.1038/s41467-017-01529-w

**Published:** 2017-11-29

**Authors:** Daniel Pérez, Ivana Gasulla, Lee Crudgington, David J. Thomson, Ali Z. Khokhar, Ke Li, Wei Cao, Goran Z. Mashanovich, José Capmany

**Affiliations:** 1 ITEAM Research Institute, Universitat Politècnica de València, Camino de Vera s/n, 46022 Valencia, Spain; 2 Optoelectronics Research Centre, University of Southampton, Highfield, Southampton SO17 1BJ UK; 3 School of Electrical Engineering, University of Belgrade, Belgrade, 11120 Serbia

*Nature Communications* **8**:636 10.1038/s41467-017-00714-1; Article published online 21 September 2017

The financial support for this Article was not fully acknowledged. The Acknowledgements should have included the following:

The fabrication was carried out in the frame of the CORNERSTONE project funded by EPSRC in the UK.

