# Supplementary material for: Multipurpose silicon photonics signal processor core
Source: Nat Commun. 2017 Sep 21;8:636. doi: 10.1038/s41467-017-00714-1 (PMC5608755; doi:10.1038/s41467-017-00714-1)
Supplement: Supplementary file 1 — Supplementary Information [file 41467_2017_714_MOESM1_ESM.pdf]

Description of Supplementary Files

File Name: Supplementary Information
Description: Supplementary Notes, Supplementary Figures, Supplementary Table and Supplementary References

File Name: Peer Review File

Supplementary Note 1: Comparison between the square- and the hexagonal- lattice mesh.

Differences between lattice mesh design topologies were addressed in a recent paper[8] including their benchmarking against a set of several figures of merit accounting for spatial tuning reconfiguration step, reconfiguration performance, number of required switching elements per unit area and losses versus spatial resolution and replication ratio.

The spatial tuning reconfiguration step identifies the ability of a waveguide mesh to synthetize a certain delay line that must be discretized in terms of fixed basic unit lengths. When the required topology to be programmed is an interferometric structure, the input and output of the delay line must fulfill certain localization requirements. In the case of an unbalanced Mach Zehnder interferometer (UMZI), both delay lines must connect the input and output couplers while maintaining the desired delay difference. For an optical ring resonator (ORR), the delay line that performs the cavity must start on one coupler side and finish in the other one. This requirement, together with the discretization of the delays limits the number of circuit topologies to be synthetized. While for Mach Zehnder interferometers (MZIs) the hexagonal mesh limits this range to a step of 2 basic unit lengths (BULs) as seen in Supplementary Equation (11), the square mesh limits are defined by a step of 4 BULs. The same figures account for cavity designs, with the exception of the minimum delay that is smaller on the square-mesh (4, 8, 12… BULs against 6, 10, 12…BULs).

In addition, as illustrated in Supplementary Figure 1 together and considering the prior argument, for the same value of ΔL, the hexagonal mesh enables a shorter (1 or 2 BUL) minimum path imbalance as compared to the minimum 3 BUL of the square mesh.

Supplementary Figure 2 illustrates that for a fixed BUL the number of available structures is greater for the hexagonal lattice mesh. For example, for a BUL = 300 µm and a maximum differential or cavity length of 10 mm, 29 and 17 basic structures are available for hexagonal and square mesh respectively. This gap increases for shorter BULs or for lager maximum differential/cavities lengths.

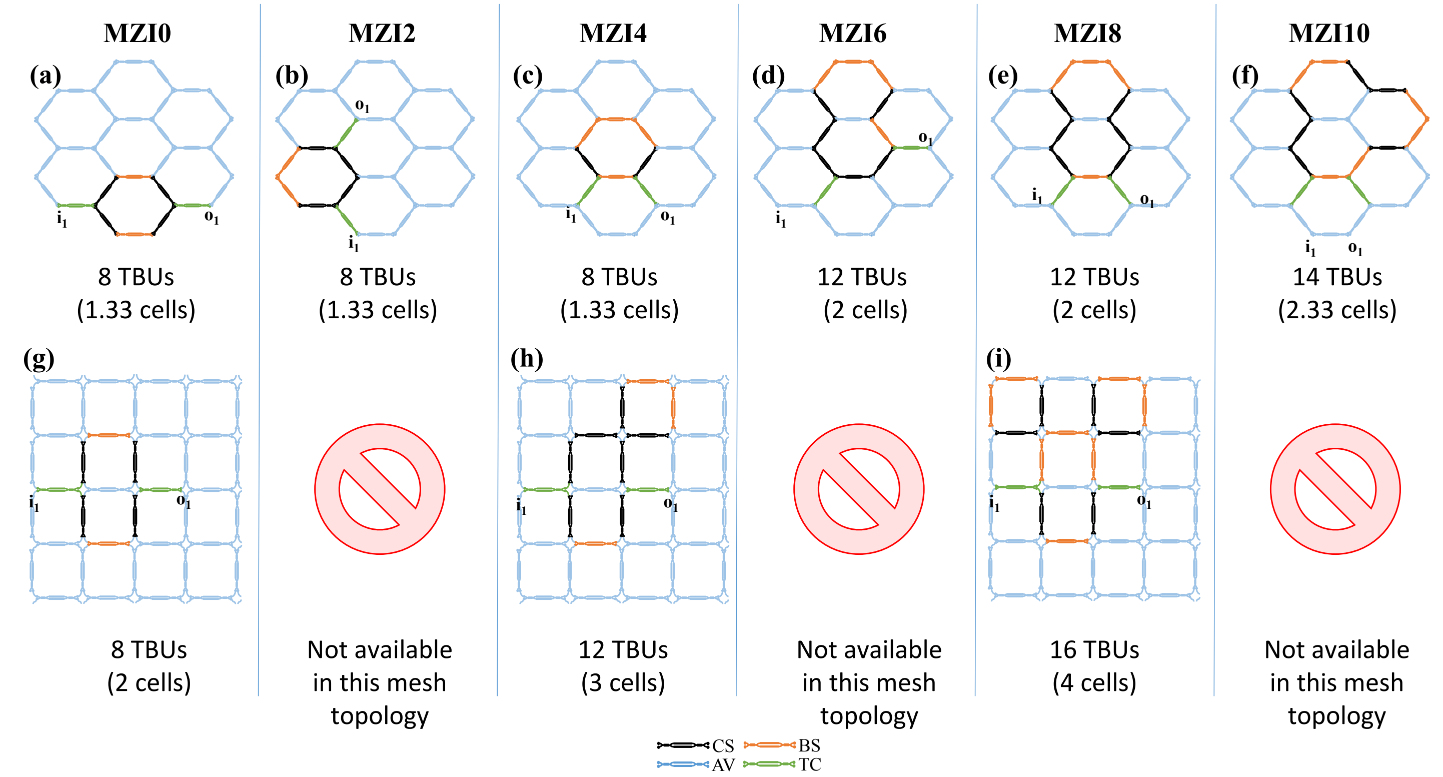

Supplementary Figure 1 | Layout for different Mach Zehnder interferometers (MZIs) implemented on a hexagonal (a)-(f) and square (g)-(i) mesh.

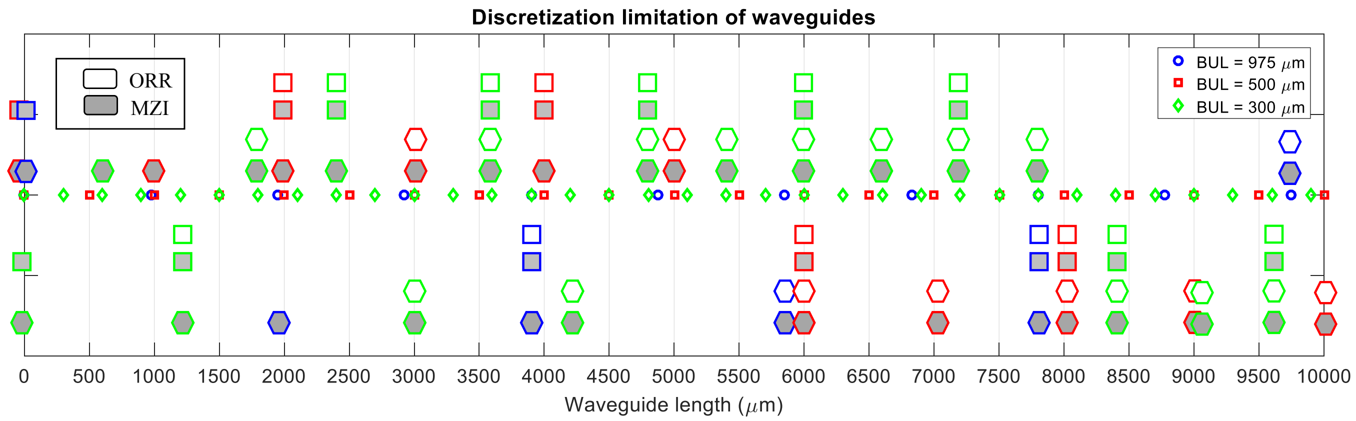

Supplementary Figure 2| Available Mach Zehnder interferometer (MZIs) or optical ring resonator (ORR) structures for hexagonal- and squared-mesh topologies for 3 different discretization values, i.e. for different basic unit lengths (BULs). The x-axis represent the differential length in the case of MZIs synthesis, and cavity length in the case of ORRs synthesis. The availability of the structure is represented by the mesh topology shape. Filled (MZI) and empty (ORR)

Supplementary Note 2: Characterization of the tuneable basic units

The tuneable basic units (TBUs) are the key building blocks of the waveguide mesh programmable photonic universal processor. As shown in Supplementary Figure 3, they are composed of 2 3-dB couplers and 2 parallel waveguides loaded with phase shifters, ϕ_{upper} and ϕ_{lower}, respectively. This MZI performs a coupler with independent amplitude and phase shifting capabilities and its ideal transfer matrix is given by:

$$h_{TBU} = je^{j\Delta}\begin{bmatrix} \sin\theta & \cos\theta \\ \cos\theta & -\sin\theta \end{bmatrix}, \quad (1)$$

where θ is (ϕ_{upper}- ϕ_{lower})/2 and Δ is (ϕ_{upper}+ ϕ_{lower})/2.

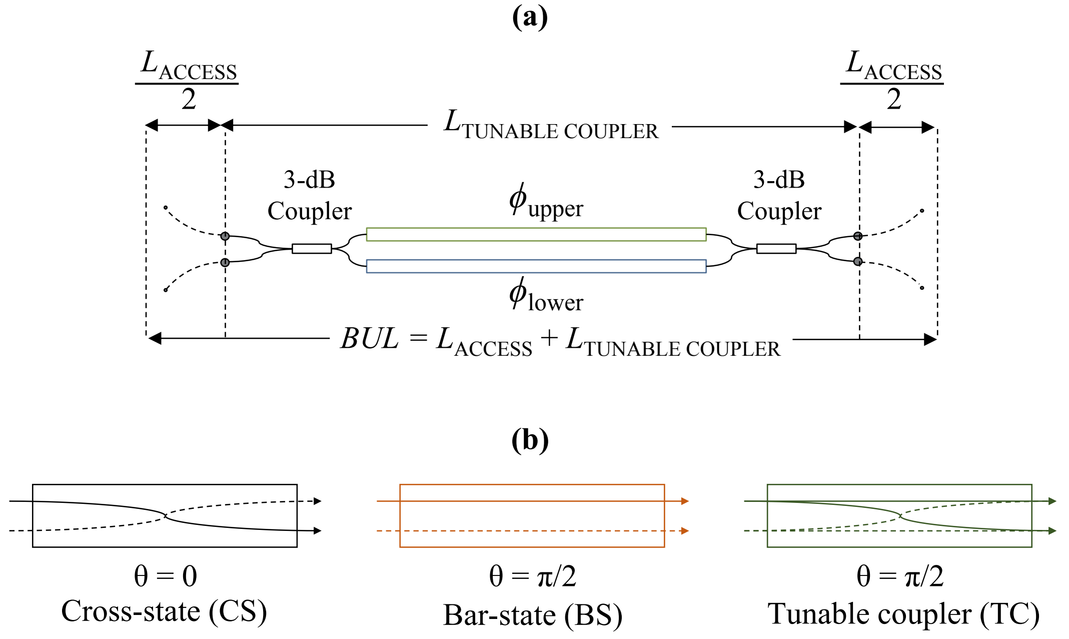

Supplementary Figure 3 | (a), Layout of a tuneable basic unit. **(b),** Configurations for different amplitude splitting ratios. Basic Unit Length (BUL).

Supplementary Figure 3 illustrates the different configurations of θ that synthetize the desired amplitude splitting ratio. The cross-state and bar-state feature a discrete delay line with a length defined by the BUL. As shown in the upper part of Supplementary Figure 1, it includes the tuneable coupler length and the access waveguides. The basic delay is given by $\tau_{TBU} = (n_g\ \mathrm{BUL})/\ c$ = 13.5 ps, where n_g is the waveguide group index and is equal to 4.18 and c is the speed of light in vacuum. In the present paper, the BUL is 975 μm. We analyse the impact and the limits of the BUL length at the end of this document.

Prior to the synthesis of any particular photonic integrated circuit (PIC), we need to characterize each one of these basic units. For each TBU that constitutes the mesh, we performed a characterization process that consisted in injecting optical power into one of

the ports of the TBU while sweeping the electrical current applied to one of the two TBU heaters. We measured the values of the output optical power and compared to the input optical power (i.e., optical losses) from its two outputs and the heater resistance. From the former measurements, we obtained the normalized coupling constant of the TBU versus the injected electrical current and power. This method also allows us to obtain the phase shift induced by the heater. The process was then repeated with the second heater of the TBU.

Supplementary Figure 4 shows, as an example, the four basic measurement curves that we obtained with this characterization step in a given TBU of the chip, rendering similar calibration curves. This process was repeated in all the TBUs that comprise the chip. The static loss measurements carried over the test unit allowed the extraction of several important material and thermal parameters that are summarized in Supplementary Table 1.

During the measurement process, we characterized four photonic dies and two complete printed circuit boards (PCBs).

Figures	Measured
Propagation Losses	2.40 ± 0.30 dB/cm
MMI Losses	0.15 ± 0.10 dB
Bend Losses	0.10 ± 0.02 dB
Grating Coupler Losses	6.50 ± 0.50 dB
Grating Coupler Central Window Deviation	1580 ± 15 nm
Grating Coupler 3-dB bandwidth	35 ± 2 nm
Thermo-optic efficiency (P_π)	110 ± 15 mW
TBU Optical Crosstalk	40 ± 5 dB
TBU Insertion Losses	0.60 ± 0.10 dB

Supplementary Table 1 | Main characterization figures.

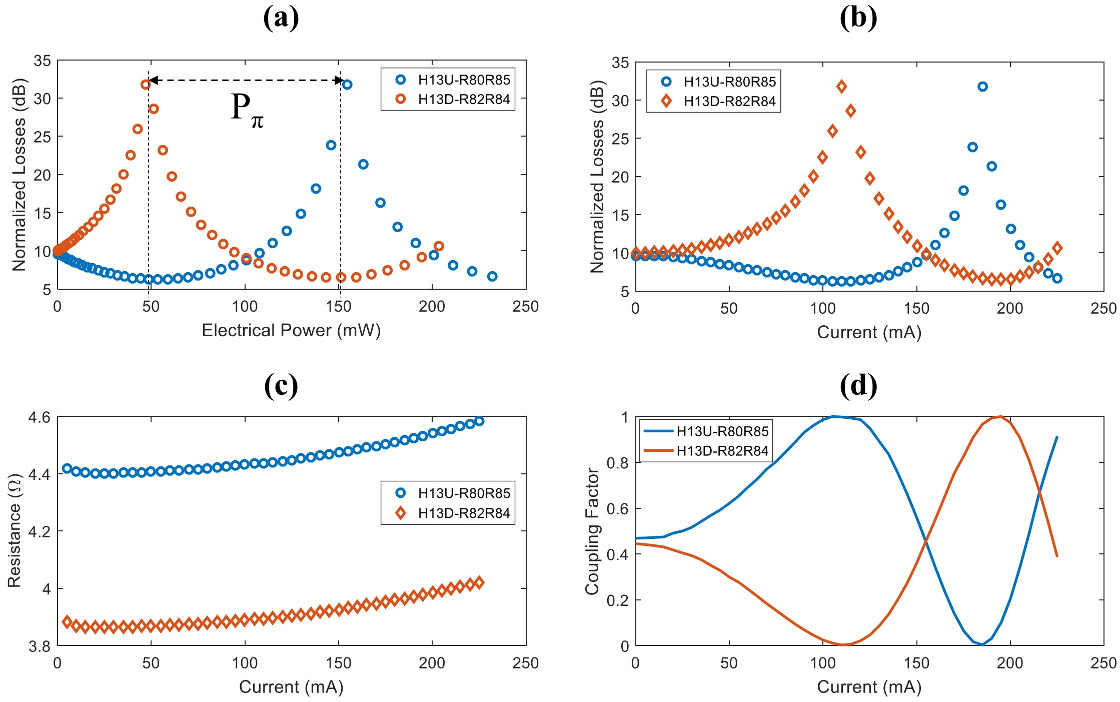

Supplementary Figure 4 | Characterization output of a tuneable basic unit. (a) Normalized losses versus electrical power applied for each heater. **(b)** Normalized losses vs electrical current. **(c)** Resistance vs electrical current. **(d)** Coupling Factor vs electrical current. PCB: Printed circuit board, TEC: Temperature in Celsius Degrees.

The TBU insertion Losses (0.60 ± 0.10 dB) include the propagation losses across the MZI and the bend relative losses. They were computed by fitting the losses of paths relative to different TBUs numbers. As a result, we extrapolated this measurement to an average synthetized waveguide loss value of 6.1 ± 0.3 dB/cm. Long-term robustness of the heaters was tested by applying an electrical power sweep up to $2P_\pi$ (over more than 2000 steps) and obtaining no-resistance variation.

tuneable

Supplementary Note 3: Adapting synthesis algorithms for universal linear interferometers to the hexagonal waveguide mesh

For Universal-Linear optics, recent papers have reported algorithms to configure arbitrary linear transformations using triangular[2,3] and rectangular[4] arrangements of beamsplitters. In this subsection, we show how these can be adapted to our proposed hexagonal waveguide mesh and, in particular, to the TBU matrix defined in the previous section.

The function of a linear optics device is to transform a series of N orthogonal modes ($|\phi_I\rangle$ into the corresponding N orthogonal modes at the output ($|\phi_O\rangle$)[2-4]. This conversion is defined by a unitary transformation matrix U ($|\phi_O\rangle = U|\phi_I\rangle$).

Miller's algorithm: The algorithm for the hexagonal waveguide mesh follows the same principles of that derived by Miller[3] with a few modifications:

First of all, we consider the possible different phase contributions due to the different access paths established between the core inputs and the internal processing elements forming the triangular arrangement of beamsplitters and, from these, to the different core outputs. These different phase contributions must be compensated. In second place, we consider a different matrix for the "beamsplitter" structure. In our case, as shown in Supplementary Figure 5, the equivalent beamsplitter is implemented using a TBU for the tuneable coupler, (with a transfer matrix defined by h_{TC} as in Supplementary Figure 1), followed by 2 TBUs, which are biased in cross state and employed as output connections. In the latter, the upper TBU also implements a phase shifter and is defined by the transfer matrix h_{UPS}. The lower TBU is defined by the transfer matrix h_{LPS}.

The original algorithm is based on writing any of the input basis functions as a linear combination of each input port or rectangular functions ($|\phi_{1n}\rangle$) (Supplementary Equation (3)), and configuring sequentially each row of beams couplers for each input mode. These input modes can be obtained from the columns of the Hermitian Adjoint of the matrix U.

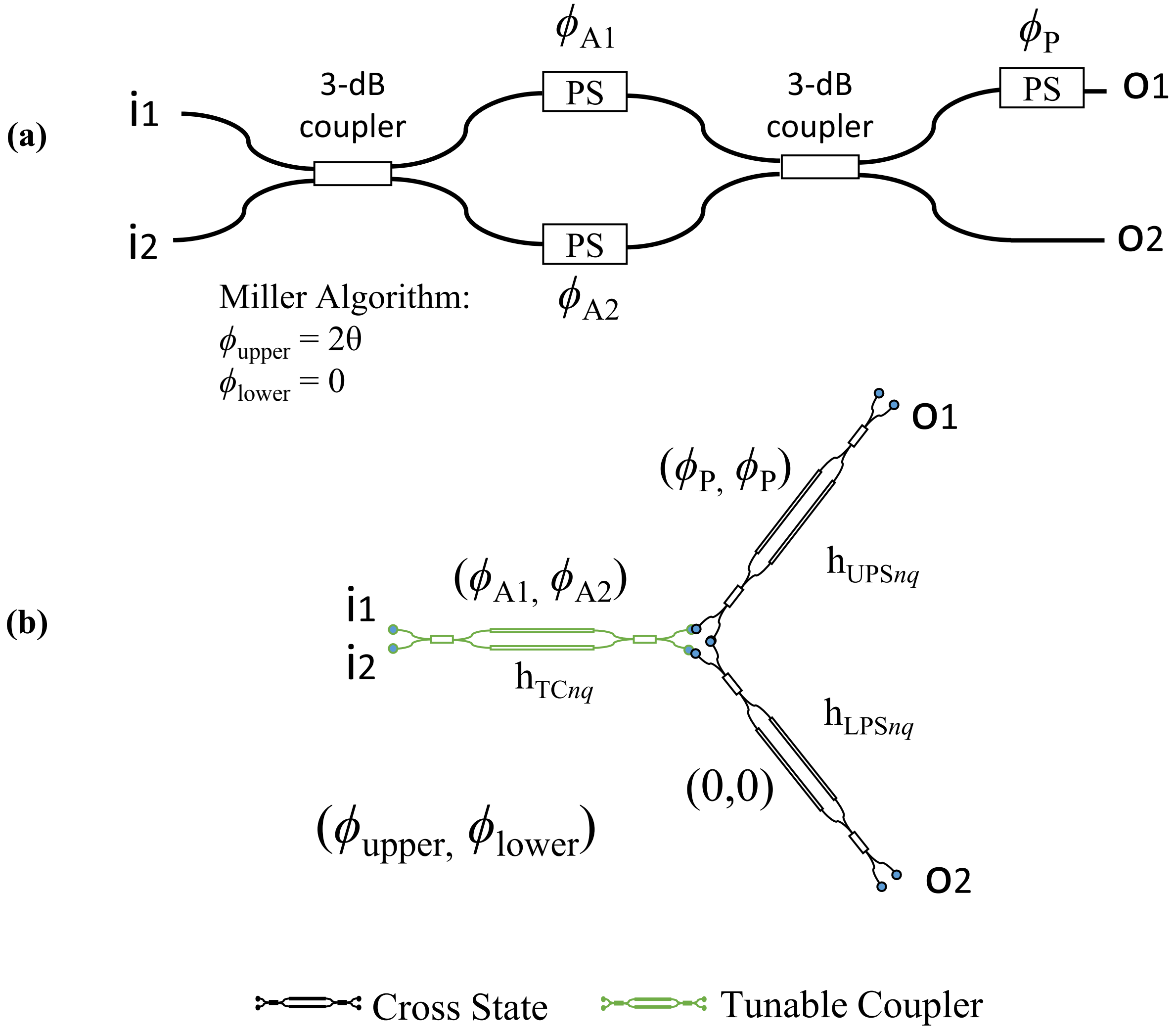

Supplementary Figure 5. Beamsplitter layout proposed for the implementation of universal linear interferometers: Miller design **(a)**, and hexagonal-mesh configurations **(b)**. PS: Phase shifter.

As mentioned, we consider first of all the impact of the access paths by multiplying the first mode by a diagonal matrix $C^{(0)}$ accounting for each input access path:

$$\left|\phi_{\mathrm{DI}1}^{(1)}\right\rangle = C^{(0)}\left|\phi_1\right\rangle, \qquad (2)$$

in $C^{(0)}$, $C^{(0)}{}_{nn}$ represents the coefficient for the n-input access path. Similarly, a $C^{(\mathrm{OutPath})}$ matrix is required to account for the output paths set after the processing elements. Supplementary Figure 5 illustrates the former concept for a 4x4 configuration. In particular, part (b) in the figure depicts 2 examples of these input and output access paths. The path responses ($C^{(0)}{}_{nn}$, $C^{(\mathrm{Outpath})}{}_{nn}$) can be computed by multiplying the proper TBU matrix element (Supplementary Equation (1)) for each of the TBUs that build up the path.

Now, we express $|\phi_{\mathrm{DI}\ 1}{}^{(1)}\rangle$ as a linear combination of each input port of the triangular arrangement of beamplitters ($|\phi_{1n}\rangle$).

$$\left|\phi_{DI1}^{(1)}\right\rangle = \sum_{n=1}^{M} a_{1n}\left|\phi_{1n}\right\rangle. \qquad (3)$$

The algorithm starts configuring the first row of beamcouplers, illustrated in Supplementary Figure 6, for the first mode. Taking into account Supplementary Equations. (2-3) and the definitions of the overall transfer matrices for the equivalent TBUs (Supplementary Figure 4), we get:

$$a_{11}^{*} = h_{\mathrm{TC}11\_11}\cdot h_{\mathrm{UPS}11\_21}\cdot c_{11}^{(\mathrm{OutPath})}. \qquad (4)$$

The algorithm first computes the modulus of $h_{\mathrm{TC}11\_11}$ satisfying Supplementary Equation (4). Note that the notation here is $h_{\mathrm{ELEMENT}qn\_xy}$, where n represents the row in the beamsplitter arrangement and q the beamcoupler order inside a given row; x and y indicate the output and input ports of the beamsplitter, respectively. Note that the modulus of the TBUs implementing phase shifters, interconnections and access paths will be 1. The phase shift of h_{UPS} is adjusted to equalize the overall phase in the left and right handside members of Supplementary Equation (4).

Once $|h_{\mathrm{TC}11\_11}|$ and the phase shift of h_{UPS} are obtained, an iterative process will render the remaining $|h_{\mathrm{TC}}|$ values and the h_{UPS} phases for the remaining beamsplitters in the row:

$$\left|h_{\mathrm{TC}1n\_11}\right|\cdot e^{i\angle\left(h_{\mathrm{UPS}1n\_21}\right)} = \frac{a_{1n}^{*}}{c_{11}^{(\mathrm{OutPath})}\prod_{q=1}^{n-1}\left|h_{\mathrm{TC}1q\_12}\right|} e^{-i\left(\angle h_{\mathrm{TC}1n\_11}+\sum_{n=1}^{n-1}\angle\left(h_{\mathrm{TC}1q\_12}\right)+\sum_{n=1}^{n-1}\angle\left(h_{\mathrm{UPS}1n\_21}\right)\right)}. \qquad (5)$$

As in the Miller algorithm, the subsequent input modes will be affected by the previously configured rows of beamsplitters. To take this fact into account, the mode has to be multiplied by the corresponding transfer function. In this case, it is defined by a triangular $C^{(n)}$ -matrix where the diagonal elements are:

$$c_{ss}^{(n)} = h_{\mathrm{TC}ns\_21}\cdot h_{\mathrm{LPS}ns\_12} \qquad (6)$$

and the upper-triangular matrix terms are:

$$c_{sj}^{(n)} = h_{\mathrm{TC}nj\_11}\cdot h_{\mathrm{TC}ns\_22}\cdot\prod_{p=s+1}^{j-1}\left(h_{\mathrm{TC}np\_12}\right)\cdot h_{\mathrm{LPS}ns\_12}\cdot\prod_{p=s+1}^{j}\left(h_{\mathrm{UPS}np\_21}\right). \qquad (7)$$

The rest of the terms of $C^{(n)}$ are 0. For the input mode n, the C matrix will incorporate $C^{(k)}$ matrices with (k = 1,2,n-1) computed for previous modes:

$$\left|\phi_{\mathrm{DI}n}^{(n)}\right\rangle = C^{(n-1)}\ldots C^{(2)}C^{(1)}C^{(0)}\left|\phi_{\mathrm{DI}n}\right\rangle. \qquad (8)$$

Some cases of special interest must be programmed apart. When the rectangular mode absolute value is $|a_{qn}|$ = 1, then all tuneable couplers $|h_{\mathrm{TC}\_11}|$ in the following equivalent beamsplitters in the row must be set to 0 except the one corresponding to the last equivalent beamplitter that will be set to 1 as well.

When the algorithm has finished, we transform the resulting values of $|h_{\mathrm{TC}\_(q,n)}|$ and the phases of h_{UPS_qn} to the actual phase shifts that have to be programmed for each TBU according to Supplementary Equation (1):

$$\begin{aligned}
\phi_{\mathrm{UPSupper}} &= \angle h_{\mathrm{UPS}} - \frac{\pi}{2},\\
\phi_{\mathrm{UPSlower}} &= \angle h_{\mathrm{UPS}} - \frac{\pi}{2},\\
\phi_{\mathrm{TCupper}} &= 2\arcsin\left(\left|h_{\mathrm{TC}qn_11}\right|\right),\\
\phi_{\mathrm{TCSlower}} &= 0,\\
\phi_{\mathrm{LPSupper}} &= 0,\\
\phi_{\mathrm{LPSlower}} &= 0.
\end{aligned} \qquad (9)$$

The algorithm that implements the configuration is the following:

```
procedure Programme (U)
    for n  from 1 to N do
        % compute |h_TC|(1,n)
        % compute ∠h_TC(1,n)
        % compute ∠h_UPS(1,n)

        if (N-I is greater than 0 then
            for q from 2 to N –i+1 do
                if (h_TC11 is equal to 1 then
                    % |h_TC11|(q:N-I,n)=0
                    % |h_TC11|(N-i+1,n)=1
                    break
                else
                    % compute |h_TC12|(q-1,n)
                    % compute |h_TC|(q,n)
                    % compute ∠h_TC(q,n)
                    % compute ∠h_UPS(q,n)
                    %compute C(n)
                    %C=C(n)…C(2)C(1)C(0)
                    %compute NextMode =
                        =C OriginalMode    Supplementary Equation (8)
                    % compute Δ, ϕ, θ Supplementary Equation (9)
```

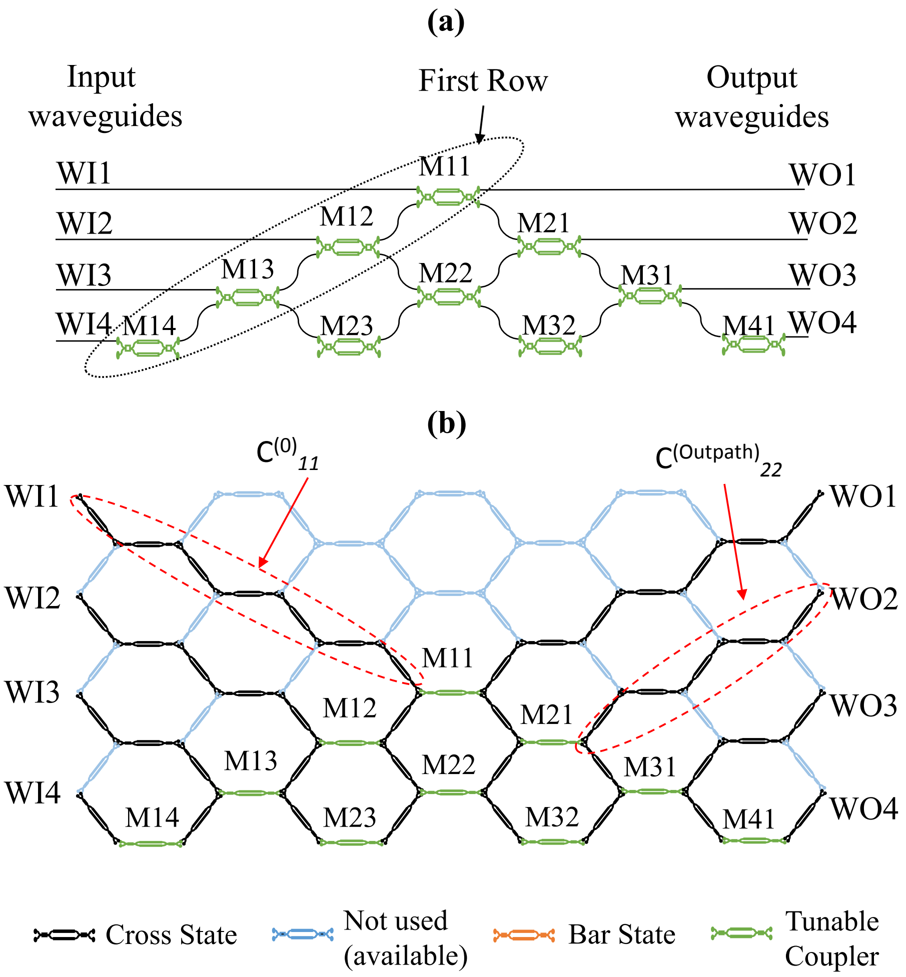

Supplementary Figure 6. Interferometer layout for unitary linear transformations: Miller design **(a)**, and hexagonal-mesh configurations **(b)**.

Clements *et al.'s* Algorithm: To adapt the synthesis algorithm proposed by Clements et al.[4] to the hexagonal waveguide mesh, we need to perform a few modifications. First of all, we use a different matrix for the "Beamcoupler/Tuneable Basic Unit (TBU)" structure. In our case, (see Supplementary Figure 7), we employ a TBU for the tuneable coupler, defined by h_{TC}, and 2 previous TBUs for the connections, where the upper one operates in cross mode providing an extra phase shifting (Upper Phase Shifter, h_{UPS}), while the lower one operates in cross mode.

Since the 2 TBUs placed before the TBU acting as a tuneable coupler are set in cross state, we can write the transfer matrix of the full beamcoupler as:

$$h_{BC} = -e^{j\Delta}\begin{bmatrix} e^{j\phi}\sin(\theta) & \cos(\theta) \\ e^{j\phi}\cos(\theta) & -\sin(\theta) \end{bmatrix} = \begin{pmatrix} e^{i\phi}r & t \\ e^{i\phi}t & -r \end{pmatrix}. \quad (10)$$

The algorithm procedure[4], consists in nulling successive matrix elements starting from the targeted unitary (NxN) matrix U. Depending on the location of the element $U(n,m)$ to be cancelled, a row or column combination of the matrix is required[4]. If N-n-m is odd, then, the element to be nulled requires a combination of columns n and m, and the updating process is done by the transformation $U'=UT^{1}_{n,m}$, while if N-n-m is even, then, the element to be nulled requires a combination of rows n, and m, and the updating process is done by the following transformation $U'=T_{n,m}U$. Here $T_{n,m}$ is a NxN identity matrix where the 2x2 submatrix formed by elements $U(n,m)$, $U(n,n+1)$, $U(m,m-1)$, $U(m,m)$ is replaced by the 2x2 matrix given in Supplementary Equation (10).

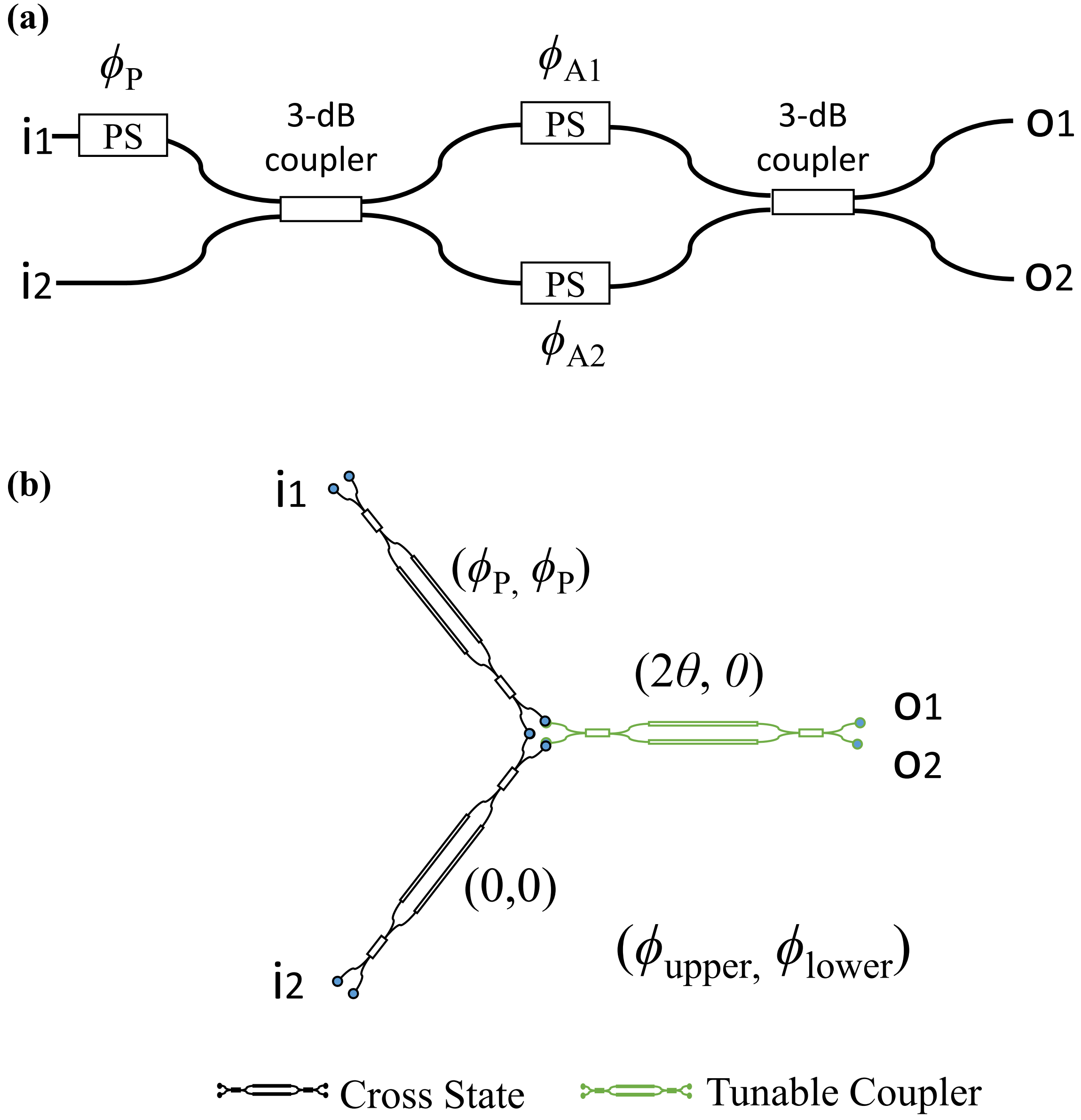

Supplementary Figure 7. Beamsplitter layout proposed for the implementation of universal linear interferometers: Clements' **(a)**, and hexagonal-mesh **(b)** configurations. PS: phase shifter.

Supplementary Note 4: Thermal stability and crosstalk

Photonic integrated circuits require, in general, thermal management. A typical strategy is to maintain the bottom of the PIC at a constant temperature for which the PIC has been designed. In our case, we have designed a copper thermal chuck coupled to a heat sink that has been employed to support a Peltier cell and a heat-sensitive resistor to perform the thermal stabilization at 28.7 ºC. This structure holds the PCB that, in turn, carries the PIC. Gold vias allow the heat to flow from the bottom to the upper part of the PCB at the PIC area.

First of all, we checked the thermal stability of our setup by synthetizing a simple optical ring resonator in the mesh and by measuring the wavelength drift of a particular spectral notch for 2 different temperatures. Supplementary Figure 8 shows that after 45 minutes a maximum deviation of less than 7 pm could be observed for both cases. In addition we obtained the temperature drift of the notch versus temperature resulting in 105 pm /ºC, which is in good agreement with typical temperature deviation figures in silicon photonics circuits[1].

Neighbouring TBUs may introduce undesired phase shift due to thermal crosstalk. The heat will flow, not only to the targeted waveguide underneath, but also to the adjacent ones producing a given phase shift. In order to minimize this effect, thermal isolation trenches were introduced in the design and fabrication to increase the thermal resistance in the waveguide plane, increasing the heat flow to the thermal sink. The results obtained for the coupling constant versus injection current seem to be reasonably immune to thermal crosstalk. We attribute this to the fact that the arms of the MZI implementing the TBU are so close that both will experience almost the same crosstalk-induced temperature variation.

Nevertheless, we noticed the presence of an extra phase shift that might be caused by 2 effects: The main one is due to the use of the same metal layer for heating and DC-signal routing. Although the DC routing tracks were made as wide as possible, only the 25% of the heating power remains in the heater area for each thermal tuner, resulting in a temperature gradient over almost all the circuit. Secondly, there might be an imperfect thermal junction between both faces of the PCB, which might need additional check and re-design. This effect is greater the closer the waveguide is from the adjacent heater, as shown in Supplementary Figure 9. Anyway, this additional phase shift can be overcome by adding a compensating additional corrective phase shift with any of the TBUs involved in the synthetized circuit.

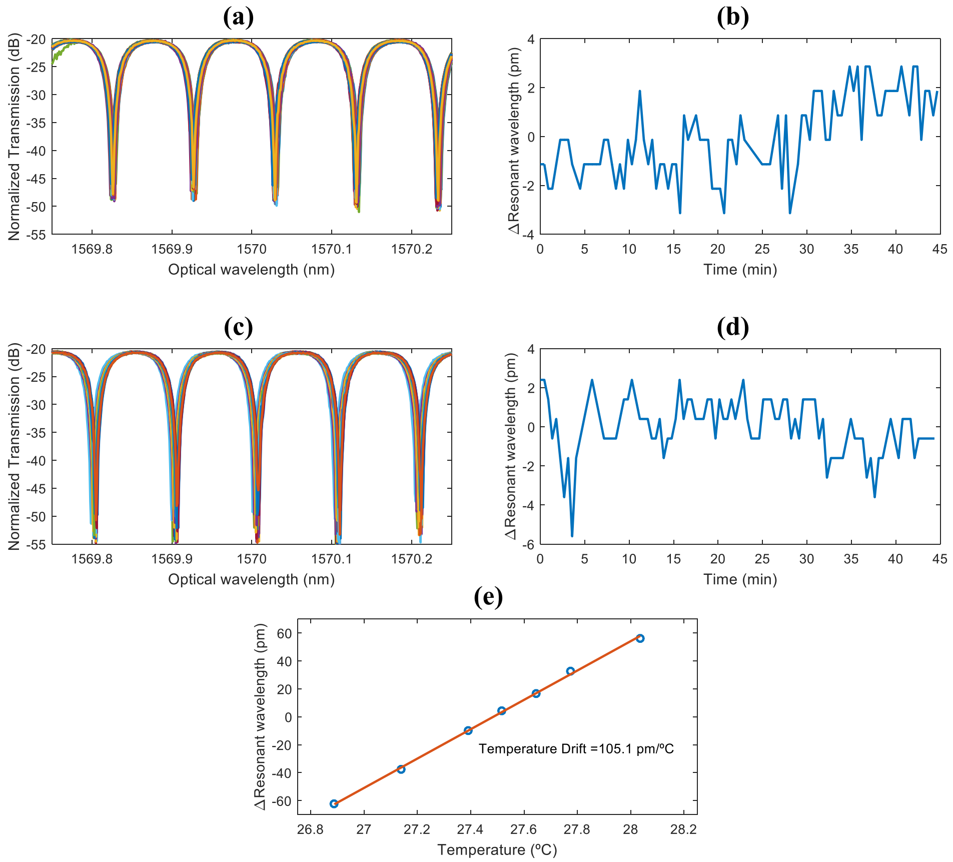

Supplementary Figure 8 | Thermal stability of the setup. (a) & (c) Normalized Transmission vs optical wavelength. **(b) & (d)** Wavelength deviation of the notches due to thermal instability. **(e)** Wavelength deviation of the notch due to the photonic integrated circuit temperature variation. ORR: optical ring resonator, TEC: Temperature in Celsius Degrees,

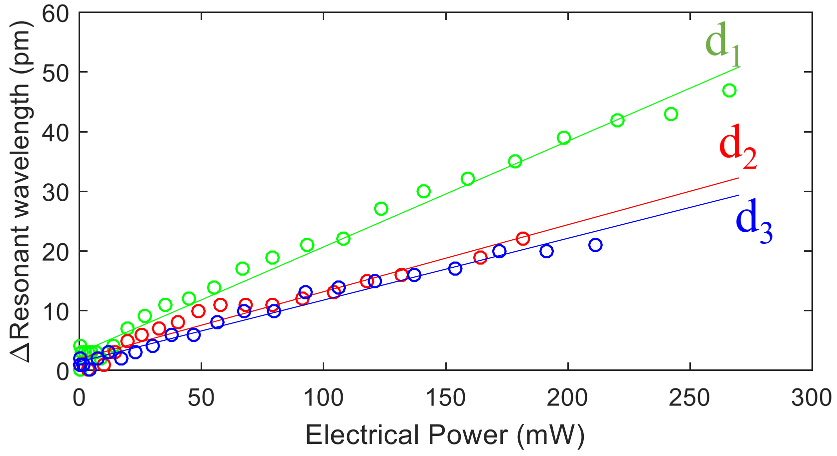

Supplementary Figure 9 | Thermal crosstalk of the setup for different distances (d_1 = 1.511, d_2 = 3.176 and d_3 = 8.876 mm) between the closest ring waveguide and the adjacent heater.

Supplementary Note 5: Full capabilities of a 7-cell honeycomb mesh

Although a 7-cell structure might appear as quite limited in terms of its capability to implement different circuits, this mesh is actually capable of supporting a considerable number of different signal processing elements. Furthermore, this capacity increases exponentially with the number of cells in the processor core. To give the reader an idea of its potential, we now provide a brief listing of identified functionalities that this 7-cell waveguide mesh can implement.

Optical Delay Lines: The fabricated 7-cell device is capable of synthetizing discrete delay lines comprising from one up to more than 30 TBUs, corresponding to a delay in excess of more than 408 ps. This building block, in combination with a continuously tuneable delay line (such as the one that can be implemented with the synthetized ring cavity), would provide larger continuously tuneable delays from 0 up to 408 ps.

Infinite Impulse Response Filters: The range of cavity lengths that can be implemented by a theoretical infinite mesh is given by[8]:

$$L_{\mathrm{ORR}}^{\mathrm{Hx}} = \{6, 10+2n\}, \quad n \in N\{0,1,2.\mathrm{K}\}. \qquad (11)$$

In the fabricated 7-cell waveguide mesh, we can synthetize single ring cavities featuring cavity lengths with the following values (normalized to a BUL): 6, 10, 12, 14, 16, 18, 20, 22, 24, 26, 28, 30, *, *, 36, *, *, up to 42 (see Supplementary Figure 10). Coupled resonator waveguides (CROWs) can couple from 2 to 7 6-BUL cavities. 10 and 12-BUL cavities CROWs can be built coupling 2 and 3 cavities. For 16- and 18-BUL cavities, only 2 resonators can be coupled. Supplementary Figure 11 shows some examples. In addition, ring cavities of different lengths (i.e., 6, 10, 12, 14, 16 or 18 BLUs) can be coupled together to exploit the Vernier effect[5].

Side-coupled integrated spaced sequences of optical resonator (SCISSOR) structures cascading 6-BUL ring cavities can be programed to cascade 2, 3, 4 or 5 units. This figure reduces to 2 when 10- or 12-BUL ring cavities are considered (see Supplementary Figure 12).

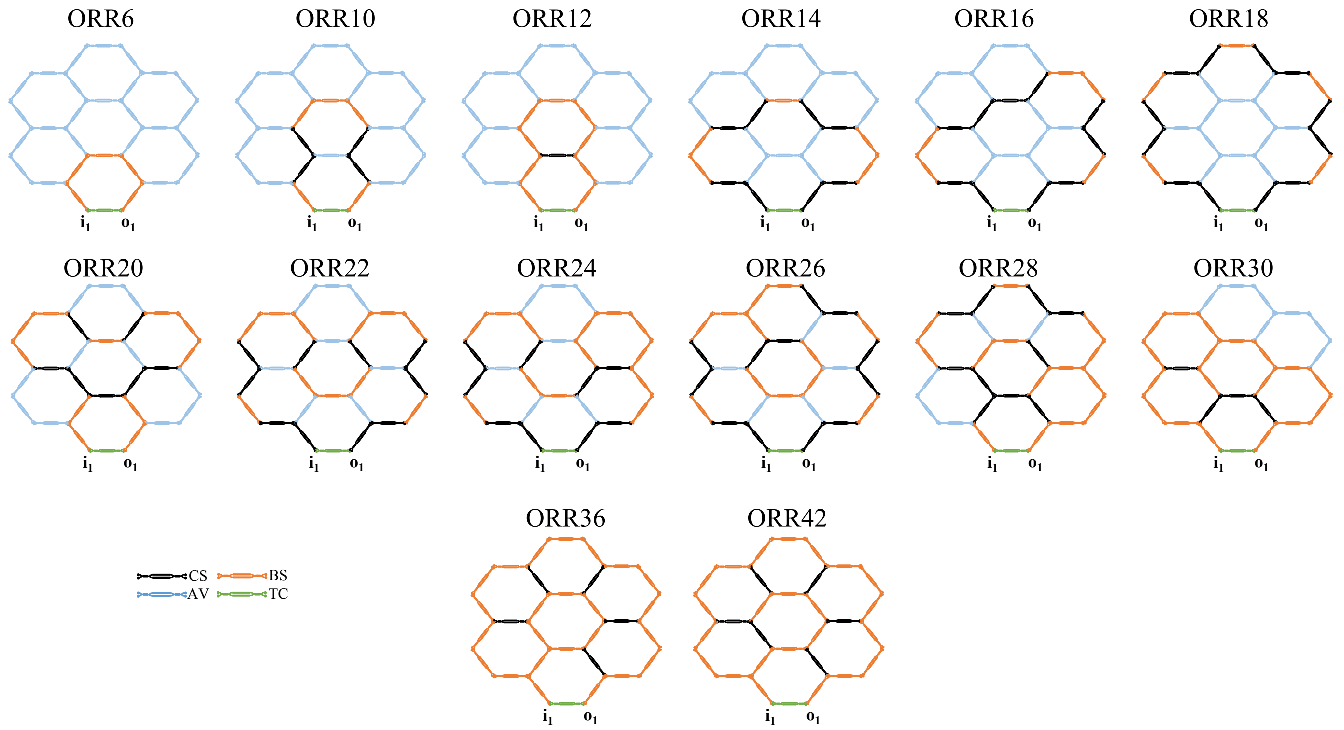

Supplementary Figure 10 | Layouts for different single ring cavity implementations. ORR: optical ring resonator, CS: cross state, BS: bar state, AV: available, TC: tuneable coupler.

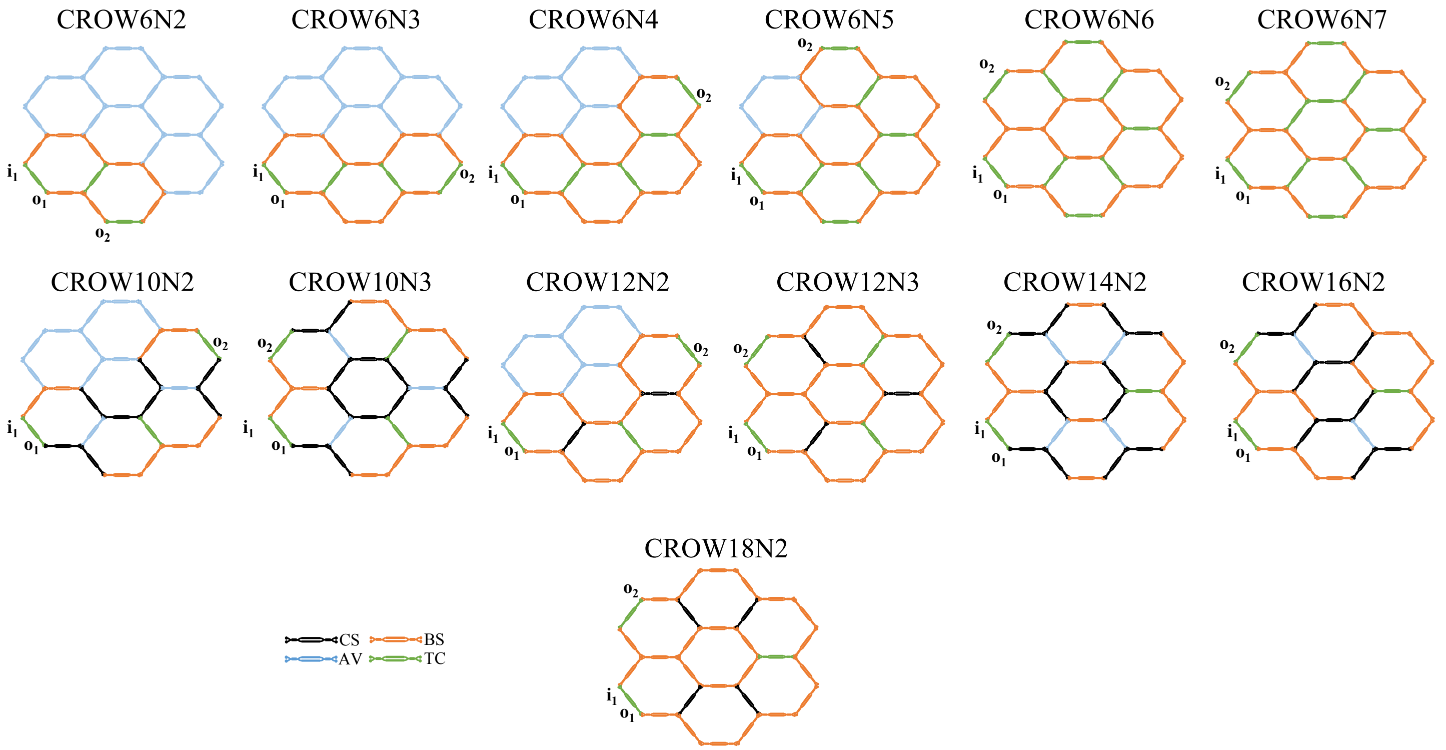

Supplementary Figure 11 | Layout for different Coupled resonator waveguide (CROW) implementations. The nomenclature is CROW *X*N*Y*, where *X* represents the cavity length (in BLUs) of the coupled ring cavities and *Y* stands for the number of coupled cavities. CS: cross state, BS: bar state, AV: available, TC: tuneable coupler.

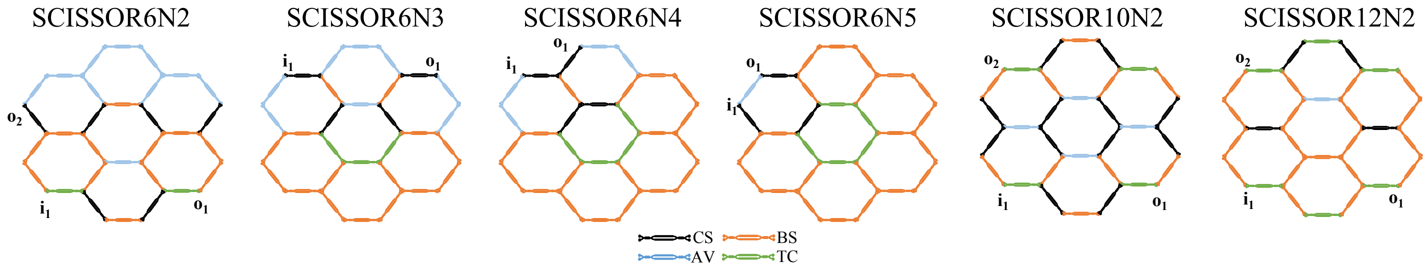

Supplementary Figure 12 | Layout for different side-coupled integrated spaced sequences of optical resonator (SCISSOR) implementations. The nomenclature is SCISSOR *X*N*Y*, where *X* represents the cavity length (in BLUs) of the coupled ring cavities and *Y* stands for the number of coupled cavities. CS: cross state, BS: bar state, AV: available, TC: tuneable coupler.

UMZI and Finite Impulse Response Filters: Single-stage UMZIs can be implemented by a theoretical infinite mesh with arm imbalances given by (in BULs)[8]:

$$\Delta L_{\text{MZI}}^{\text{Hx}} = 2n \qquad n \in N\left\{0,1,2.K\right\} \qquad (12)$$

In the 7-cell case, the former equation renders the following values: 0, 2, 4, 6, 8, 10, 12, 14, 16 and 18 BULs (see Supplementary Figure 13). A remarkable property of the hexagonal mesh is that it can implement a 2-BUL UMZI. This is not possible with square or triangular mesh designs[3]. In our particular chip this structure yields a spectral period of 36.8 GHz. Transversal filters with more than 2 taps can be also programmed as demonstrated in the main text.

Complex Ring-Loaded UMZI filters: As we outlined in the main text, we can program the waveguide mesh to implement filters that are more complex by combining different basic UMZI and Ring cavity units (see Supplementary Figure 14). The 7-cell fabricated device allows, for instance, for the realization of a UMZI loaded with a ring filter with cavity lengths in the following range (in BUL units) 6, 10, 12, 14, 18, and 24. Also, the load can be a CROW of 2 coupled 6-BUL ring resonators. Finally, both arms can be loaded with independent ring resonators of 6 BULs.

Universal Linear Interferometers: Probably, the most powerful feature of the waveguide mesh lies in its potential to implement linear optics transformations. These are given by unitary *N*x*N* matrices that relate *N* input modes to *N* output modes. In the case of the waveguide mesh, there is a straightforward correspondence between input/output modes and the physically different spatial inputs/outputs of the devices. Regarding our 7-cell structure, we can program any linear operation defined by a 2x2, 3x3 or 4x4 matrix programmed. Reconfigurable linear optics transformations play a fundamental role in classical systems where they can be exploited for routing, switching and system interconnections[3]. More recently, these operations have been exploited in the realm of quantum information systems where they play a fundamental role in the implementation of quantum logic gates[9]. Our 7-cell mesh can be programmed to implement linear transformations that can be employed to program popular quantum logic 2x2 gates such as Pauli X, Y and Z, and Hadamard, but also more complex functionalities, such as the C-NOT, C-PauliX, C-PauliY and C-PauliZ gates (a universal gate for quantum circuits) and the SWAP gate. Supplementary Figure 15 shows some examples.

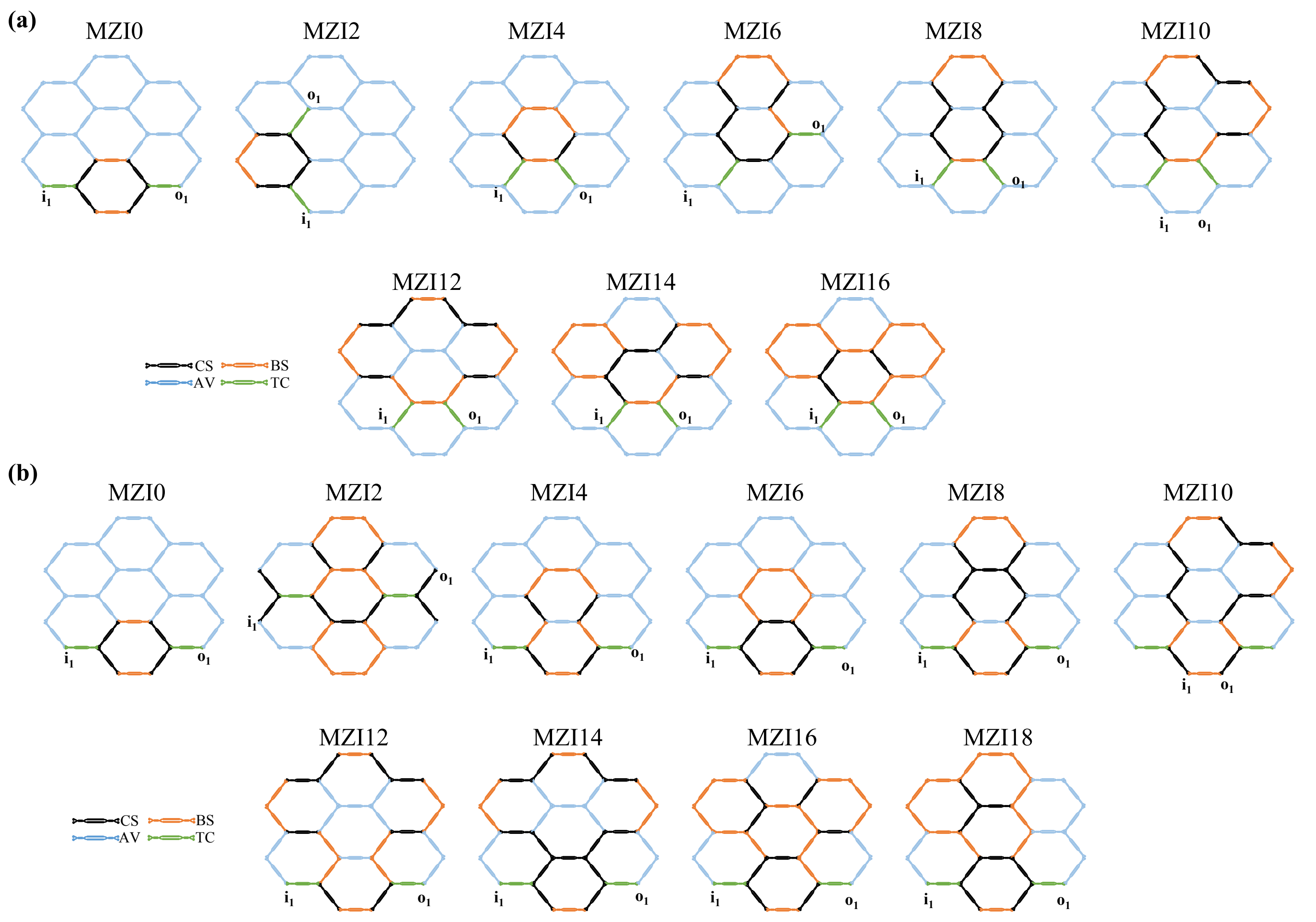

Supplementary Figure 13 | Layout for different Mach Zenhder interferometer (MZI) implementations. a) lower insertion losses implementation and **b)** constant 3-BUL shorter path implementation. The nomenclature is MZI*X*, where *X* represents the length unbalance between the two arms in BUL units. Larger BULs are possible by using a shorter reference length as in Supplementary Figure 4. Basic Unit Length (BUL), CS: cross state, BS: bar state, AV: available, TC: tuneable coupler.

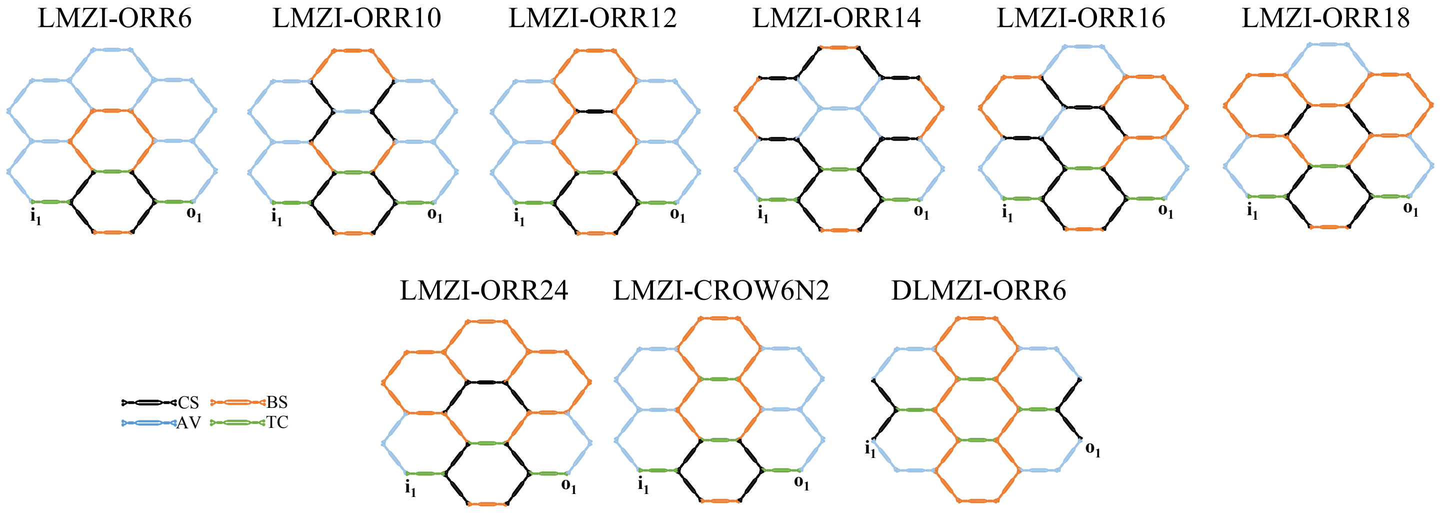

Supplementary Figure 14 | Layouts for different loaded Mach Zenhder interferometer (MZI) implementations. The Nomenclature is *A*MZI-*BX*N*Y*, where *A* = *L* represents a single arm loading, *A* = *D* a double arm loading, *B* represents the load, *B* = ORR represents an optical ring cavity (ORR), *B* = CROW represents a Coupled resonator waveguide (CROW) filter, *X* represents the cavity length (in BLUs) of the coupled ring cavities and *Y* stands for the number of coupled cavities. Basic Unit Length (BUL), CS: cross state, BS: bar state, AV: available, TC: tuneable coupler.

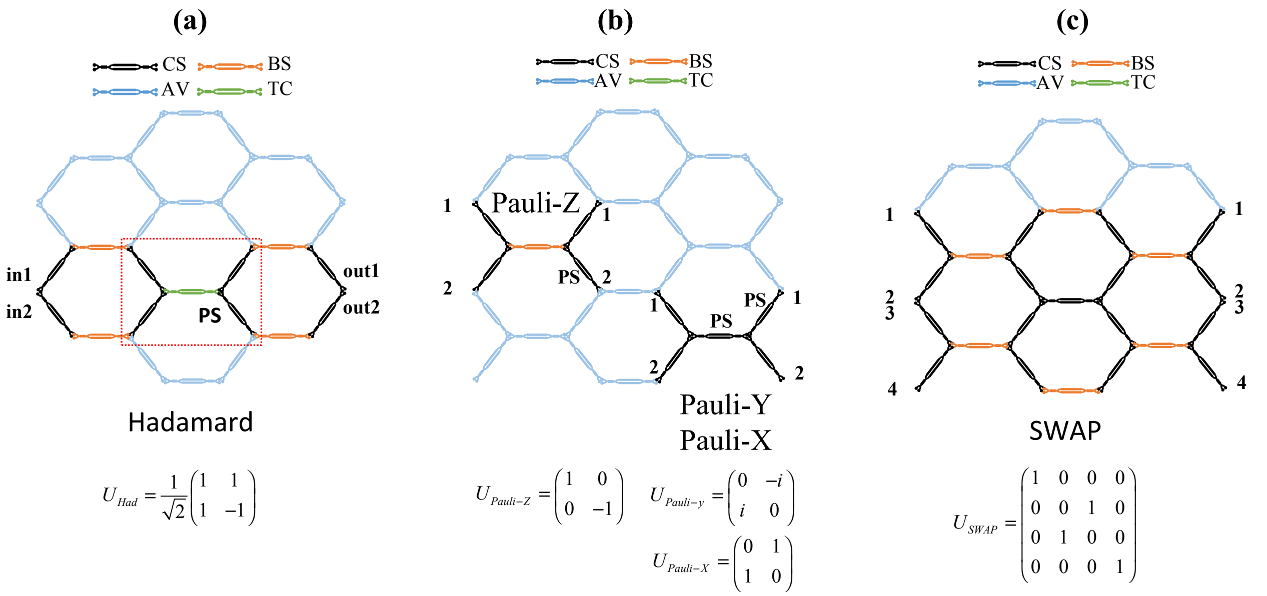

Supplementary Figure 15 | Layout for different compact 2x2 and 4x4 quantum logic gates. (a) Hadamard, (b) Pauli-Y and Pauli-X, (c) SWAP. CS: cross state, BS: bar state, AV: available, TC: tuneable coupler.

Supplementary Note 6: Implementation and characterization of Photonic Integrated Circuits

We implemented over 20 different circuit configurations by tuning the pertinent TBUs using up to 18 current sources (3 Keihtley 2401, 13 Thorlabs LDC8010 and 2 TECMA 72-2535 models). This restriction in the available number of current sources, and not the mesh size, actually limited the number of circuits we could demonstrate experimentally.

To characterize the optical responses as a function of the wavelength, we employed an ANDO AQ6217C optical spectrum analyzer together with a tuneable laser ANDO AQ4321D, which featured an optical resolution of 1 pm.

The proposed architecture can implement both traditional feedforward/feedbackward FIR and IIR filters as well as universal multiple input/multiple output optical linear transformers. FIR filters are based either on cascades/lattices of 3-dB tuneable MZIs or transversal filter configurations[5,6]. For both FIR filter alternatives, synthesis and recursive scaling algorithms have been developed in the literature[5,6] that are directly applicable since the hexagonal waveguide mesh can implement both 3-dB tuneable MZI cascade lattices and transversal filter configurations. For IIR filters, either simple/compound optical ring cavities of ring-loaded 3-dB tuneable MZI cascades are employed[5,7]. Again, synthesis algorithms have been reported in the literature[5,7] that are directly applicable since the hexagonal waveguide mesh can implement either simple or multiple cavity ring filters or ring-loaded 3–dB tuneable MZI cascades.

We compared each measured case to its well-known analytical transfer function that can be derived following the methods described[5-7] and obtained an excellent match for both amplitude and phase responses. For example, in the case of the tuneable 3-dB MZI structure, the analytical transfer function connecting input port 1 and output port 2 is given by:

$$h_{21}^{\mathrm{NMZI}}(\lambda) = j\sqrt{(1-\gamma_{\mathrm{a}})(1-\gamma_{\mathrm{b}})}\,e^{-j\beta_{\mathrm{d}}^{(\mathrm{P})}(\lambda)L_{\mathrm{d}}^{(\mathrm{P})}}\left[\sqrt{K_{\mathrm{b}}^{(\mathrm{P})}(\lambda)\left(1-K_{\mathrm{a}}^{(\mathrm{P})}(\lambda)\right)}\,e^{-j\Delta\phi_{\mathrm{ud}}^{(\mathrm{P})}}+\sqrt{K_{\mathrm{a}}^{(\mathrm{P})}(\lambda)\left(1-K_{\mathrm{b}}^{(\mathrm{P})}(\lambda)\right)}\right], \qquad (13)$$

where

$$\Delta\phi_{\mathrm{ud}} = \beta_{\mathrm{u}}^{(\mathrm{P})}L_{\mathrm{u}}^{(\mathrm{P})} - \beta_{\mathrm{d}}^{(\mathrm{P})}L_{\mathrm{d}}^{(\mathrm{P})} = \beta_{\mathrm{u}}^{(\mathrm{P})}\cdot\left(n_{\mathrm{u}} - \Delta\beta_{\mathrm{ud}}^{(\mathrm{P})}n_{\mathrm{d}}\right)\cdot BUL, \quad \left(|n_{\mathrm{u}} - n_{\mathrm{d}}| = N\right). \qquad (14)$$

The parameters $\gamma_{\mathrm{a,b}}$ and $K_{\mathrm{a,b}}^{(\mathrm{P})}$ represent the excess losses and coupling factor of the input and output couplers in the MZI structure respectively, $\beta_{\mathrm{u,d}}$ the propagation coefficient of the upper and lower waveguide, respectively, and $L_{\mathrm{u,d}}$ the length of the upper and lower paths. In the case of the ring cavity structure, the reflection and transmission transfer functions are given by:

$$h_{\mathrm{ref}}^{\mathrm{NORR}}(\lambda) = \sqrt{(1-\gamma)\left(1-K^{(\mathrm{P})}(\lambda)\right)}\cdot\frac{1-(1-\gamma)e^{-j\beta^{(\mathrm{P})}(\lambda)L^{(\mathrm{P})}}}{1-(1-\gamma)\left(1-K^{(\mathrm{P})}(\lambda)\right)e^{-j\beta^{(\mathrm{P})}(\lambda)L^{(\mathrm{P})}}},$$
$$h_{\mathrm{trans}}^{\mathrm{NORR}}(\lambda) = \frac{-(1-\gamma)K^{(\mathrm{P})}(\lambda)e^{-j\beta^{(\mathrm{P})}(\lambda)L^{(\mathrm{P})}/2}}{1-(1-\gamma)\left(1-K^{(\mathrm{P})}(\lambda)\right)e^{-j\beta^{(\mathrm{P})}(\lambda)L^{(\mathrm{P})}}}, \qquad (15)$$

where

$$\beta^{(\mathrm{P})}L^{(\mathrm{P})} = \beta^{(\mathrm{P})} \cdot N \cdot BUL. \qquad (16)$$

The parameters γ and $K^{(\mathrm{P})}$ represent the excess loss and coupling factor of the ORR coupler respectively. The superscript (P) indicates that this parameter can be tuned in the TBU.

Supplementary Figure 16 shows, as an example, the results for two different filters, a 4-BUL UMZI and a 6-BUL ring cavity filter. For the UMZI, we configured and simulated $K_1 = 0.5$, while K_2 took the values 0.11 (yellow), 0.26 (red) and 0.34 (blue). For the ring cavity, we selected $K_2 = 0$ while K_1 took the values 0.15 (yellow), 0.50 (red) and 0.65 (blue). Coupler positions can be identified in Figs. 3 and 4 in the main manuscript.

Supplementary Figures 17-19 provide the experimental results of additional UMZI, ring cavity and complex FIR + IIR structures that were implemented with the waveguide mesh processor subject to the limitation of the available 18 current sources (for the sake of simplicity, we only include the transfer function modulus). Supplementary Figure 17(a) illustrates the implementation of a 2-BUL UMZI featuring a 36.8-GHz FSR. Supplementary Figure 17(b) demonstrates the phase-shifting capabilities of a single TBU by moving the zero of the synthetized 2-BUL UMZI along the complex plane and thus tuning the resonance a complete FSR. Supplementary Figures 17(c) and 17(d) correspond to a 4-BUL UMZI.

The ring cavity filter results in Supplementary Figure 18 illustrate two concepts. The first one is the setting of one of the TBUs in the mesh to act as cross switch allowing the simultaneous use of its 4 ports to increase a given path length. This is shown in Supplementary Figure 18(a), where we programmed a 12-BUL ring cavity filter employing 11 TBUs. This element can be employed as well in bar configuration to save area and used TBUs at the risk of adding optical crosstalk. The second interesting concept is the ability of implementing long cavities with practically no crosstalk by routing the light through the waveguides of the outer perimeter in the mesh. In this way, we could implement an 18-BUL ring cavity filter with 18 TBUs. The results are shown in Supplementary Figure 18(b).

Other filter structures with more complex layouts were programmed as well. Supplementary Figure 19(a) illustrates a CROW structure where two cascaded ring resonators (input: IN, output: OUTPUT 1) implement a series of reconfigurable filters by arbitrarily moving its zeros and poles. One of the TBUs identified by an asterisk (*) was kept unbiased. Nevertheless, this is an example of how TBUs can be configured in order to extract non-ideal leaking due to optical crosstalk from the circuit. Supplementary Figure 19(b) shows the reflection response of two coupled ring resonators in a SCISSOR configuration (input: IN, output: OUTPUT 1). This circuit structure is widely used for dispersion compensation and reconfigurable filtering. Supplementary Figure 19(c) shows the transmission response (input: IN, output: OUTPUT 2) of a CROW comprising three ring resonators.

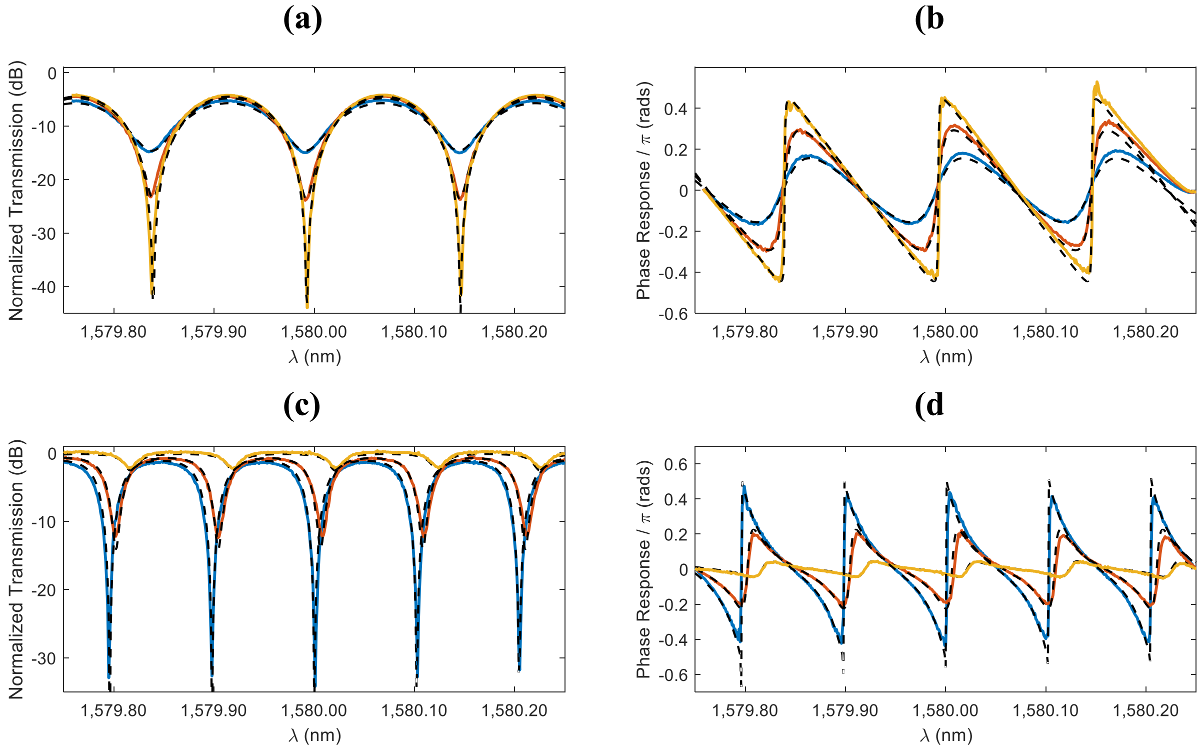

Supplementary Figure 16 | Comparison between simulated and measured filters. (a) Power and **(b)** phase responses of a Mach Zehnder interferometer (MZI), **(c)** Power and **(d)** phase responses of an optical ring resonator (ORR). Dashed lines correspond to simulated values whereas solid lines correspond to measured traces.

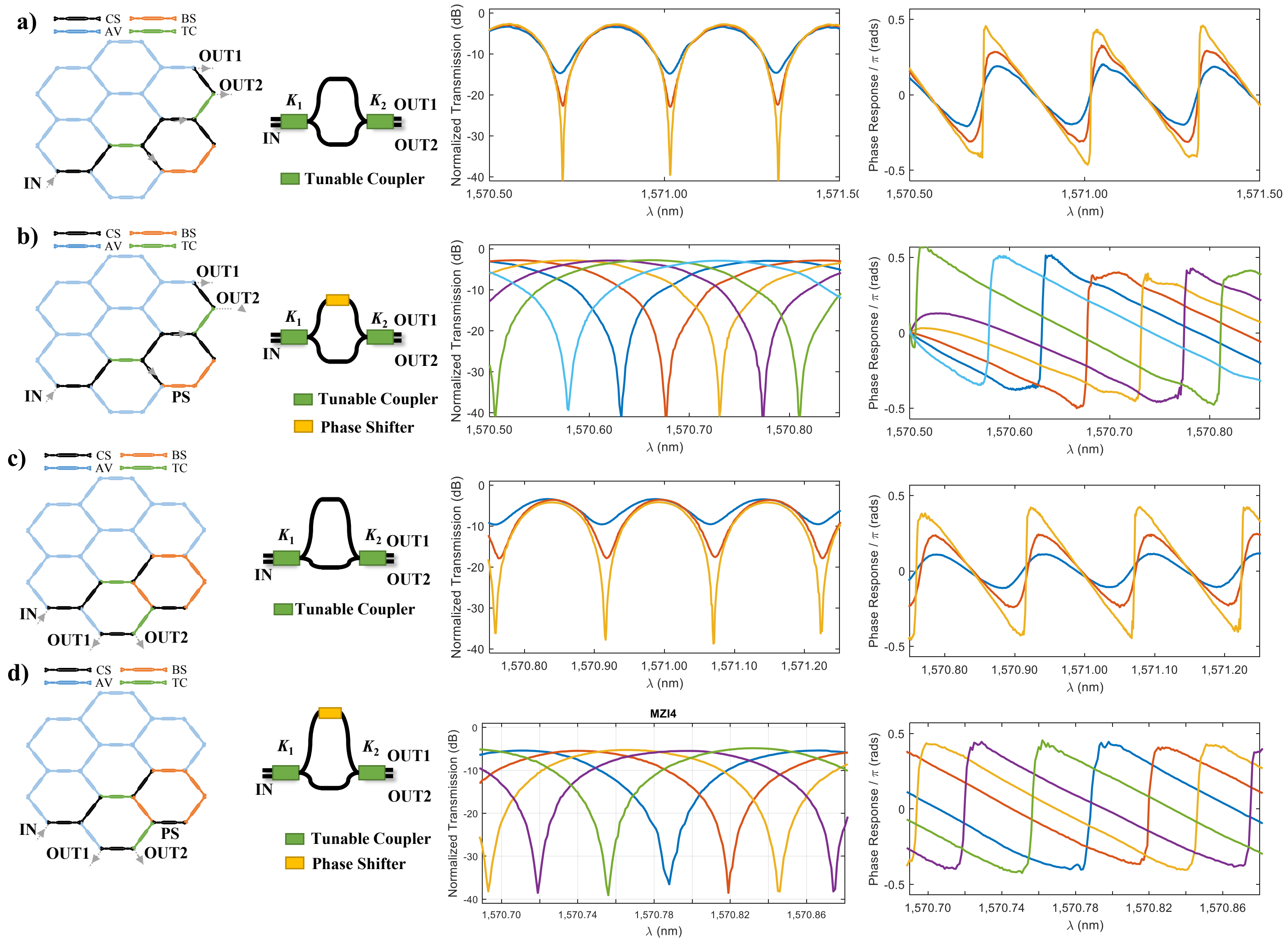

Supplementary Figure 17 | Experimental results for tuneable unbalanced Mach Zehnder interferometers (UMZIs). Waveguide mesh connection diagram, circuit layout and measured modulus and phase transfer function for different values of the coupling constants K_1 and K_2 in the case of **a** and **b,** a 2-BUL UMZI filter; **b** and **c,** a 4-BUL UMZI filter. For examples **b** and **d**, K_1 and K_2 remain fixed and the phase shift value is applied to provide full FSR tuning. For each case, the first column shows the 7-cell hexagonal waveguide mesh configuration, where each Mach Zehnder Interferometer (MZI) device is represented by a given colour depending on whether it is activated as a cross (black) or bar (orange) switch, a tuneable coupler (green) or not used/available (blue). The second column shows the layout of the implemented structure, while the third column shows the measured modulus for the synthesized configuration where the input is in the IN port and the output is the OUT1 port. BUL: basic unit length, PS: phase shifter, CS: cross state, BS: bar state, AV: available, TC: tuneable coupler.

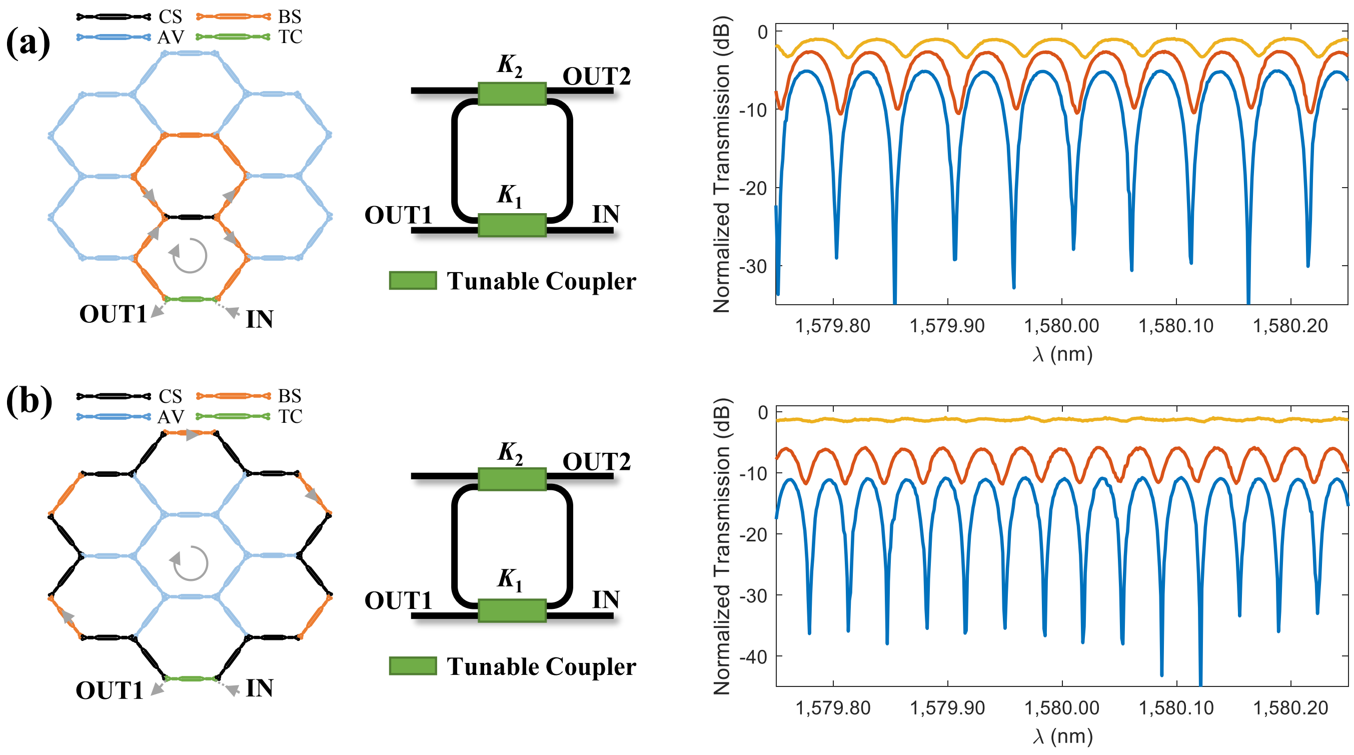

Supplementary Figure 18 | Experimental results for tuneable Ring cavities. Waveguide mesh connection diagram, circuit layout and measured modulus for different values of the coupling constants K_1 and K_2 in the case of **a,** a 12-BUL optical ring resonator (ORR) filter; **b,** an 18-BUL ORR filter. For each case, the first column shows the 7-cell hexagonal waveguide mesh configuration, where each Mach Zehnder Interferometer (MZI) device is represented by a given colour depending on whether it is activated as a cross (black) or bar (orange) switch, a tuneable coupler (green) or not used/available (blue). The second column shows the layout of the implemented structure, while the third column shows the measured modulus for the synthesized configuration where the input is in the IN port and the outputs are the OUT1 and OUT2 ports. BUL: basic unit length, CS: cross state, BS: bar state, AV: available, TC: tuneable coupler.

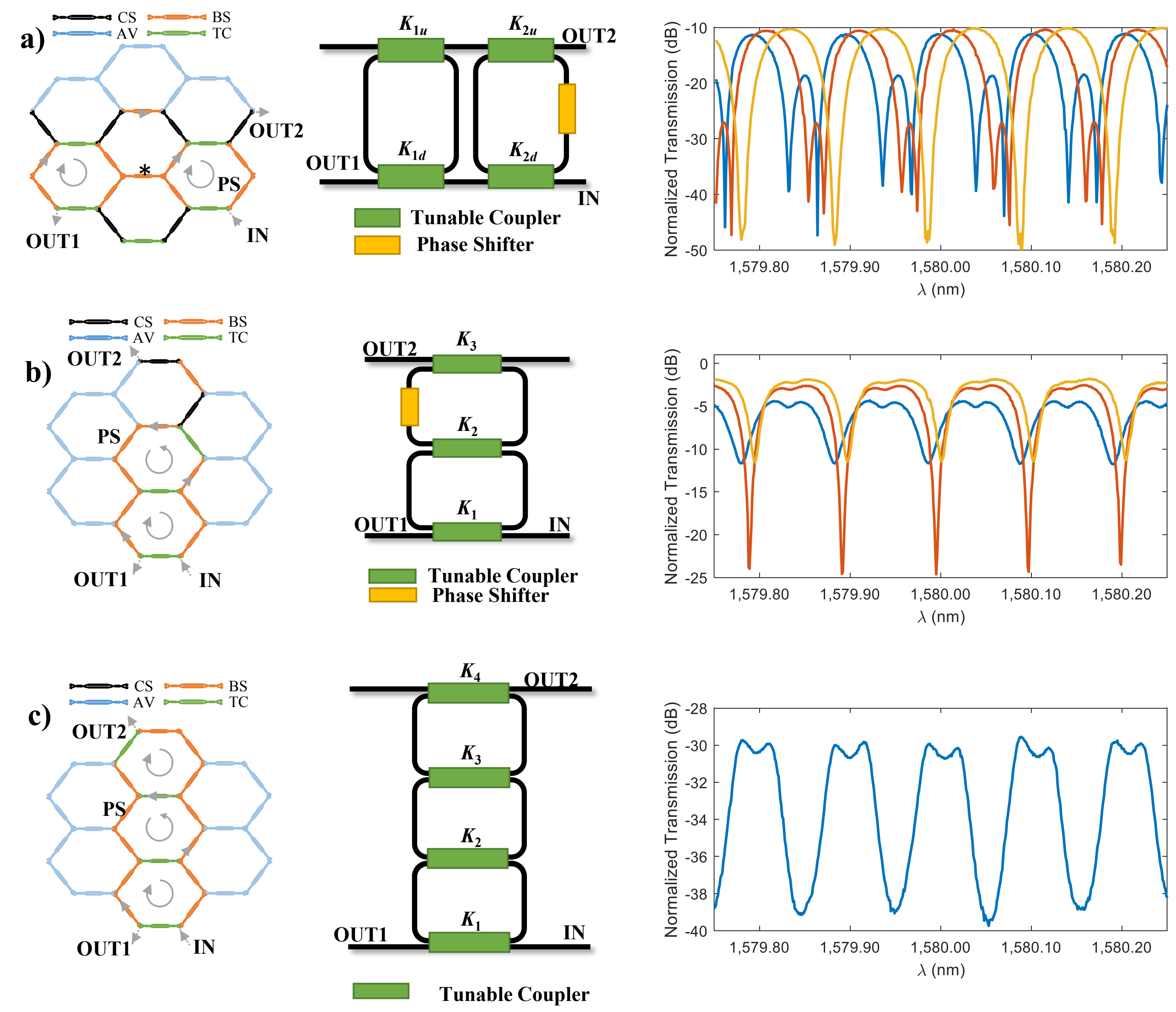

Supplementary Figure 19 | Experimental results for tuneable unbalanced Mach Zehnder interferometers UMZIs and finite impulse response (FIR) Filters. Waveguide mesh connection diagram, circuit layout and measured modulus for different values of the coupling constants K_1 and K_2 in the case of **a),** a 6-BUL side-coupled integrated spaced sequences of optical resonator (SCISSOR) filter; **b),** a 6-BUL Coupled resonator waveguide (CROW) filter; **c),** a 6-BUL CROW. For each case, the first column shows the 7-cell hexagonal waveguide mesh configuration, where each Mach Zehnder Interferometer (MZI) device is represented by a given colour depending on whether it is activated as a cross (black) or bar (orange) switch, a tuneable coupler (green) or not used/available (blue). The second column shows the layout of the implemented structure, while the third column shows the measured modulus for the synthesized configuration where the input is in the IN port and the outputs are the OUT1 and OUT2 ports. BUL: basic unit length, PS: phase shifter, CS: cross state, BS: bar state, AV: available, TC: tuneable coupler.

Supplementary Note 7: Mesh scalability limits

There is a strong relationship between the number of cells and the number of TBUs and the BULs.

The impact of the BUL is twofold: First, it sets the minimum resolution step defined in the previous section. When a BUL tends to 0, the mesh resolution will be finer and arbitrary designs will fit in better within the mesh. In addition, the BUL is inversely proportional to the maximum Free Spectral Range of the frequency periodic interferometric structures, which will determine the maximum operation frequency range for microwave photonics operations[8].

In second place, a trade-off appears in term of losses. Supplementary Figure 20 illustrates the impact of the multimode interferometer (MMI) insertion losses and waveguide propagation losses in the layout depicted in Supplementary Figure 1 for 3 different discretization (BUL = 300, 500 and 975 μm) values. The minimum number of TBUs required to provide a 1-cm waveguide is 11 for the larger BUL and 34 for the shorter one. We can observe that the MMI insertion losses are the most limiting factor, hence a higher discretization leads to higher losses. State-of-the art MMIs insertion and propagation losses on silicon photonics are around 0.1

dB/MMI and 1 dB/cm, respectively, enabling reduced-loss programmable waveguides. It is worth noting as well that reduced BULs would lead to an increase in the thermal crosstalk due to the tuning mechanism.

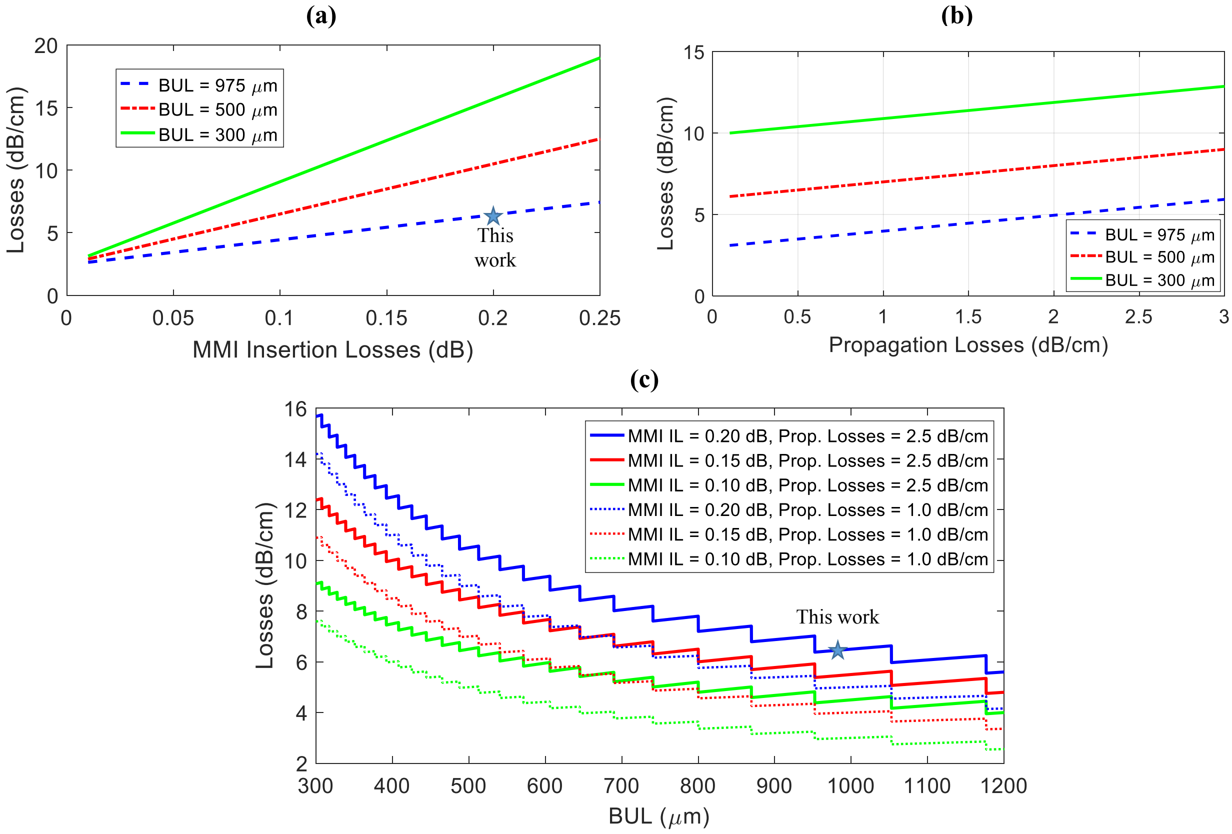

Supplementary Figure 20. Synthetized waveguide losses impact due to. **(a)** multimode interferometer (MMI) Insertion Losses (IL) for propagation losses of 2.5 dB/cm. **(b)** propagation losses for a fixed MMI IL of 0.15 dB. **(c)** discretization due to Basic Unit Length (BUL) variation.

The number of cells for a particular design will depend on the size of the targeted circuits to be synthetized/programmed. In practice, the number of electrical DC Pads and its associated control system might limit the number of available TBUs present on the circuit. Supplementary Figure 21 illustrates the number of electrical ports versus the number of cells. In the present paper, we employ 120 single-point (ground) pads for the electrical control of 60 phase shifters. Out of the chip, some of them were connected to a common ground. Having a common ground port on the circuit can reduce the amount of electrical ports by a factor of 2. The main drawback would then be the appearance of electrical noise, which can be pre-characterized considering the differential resistance of each path[10].

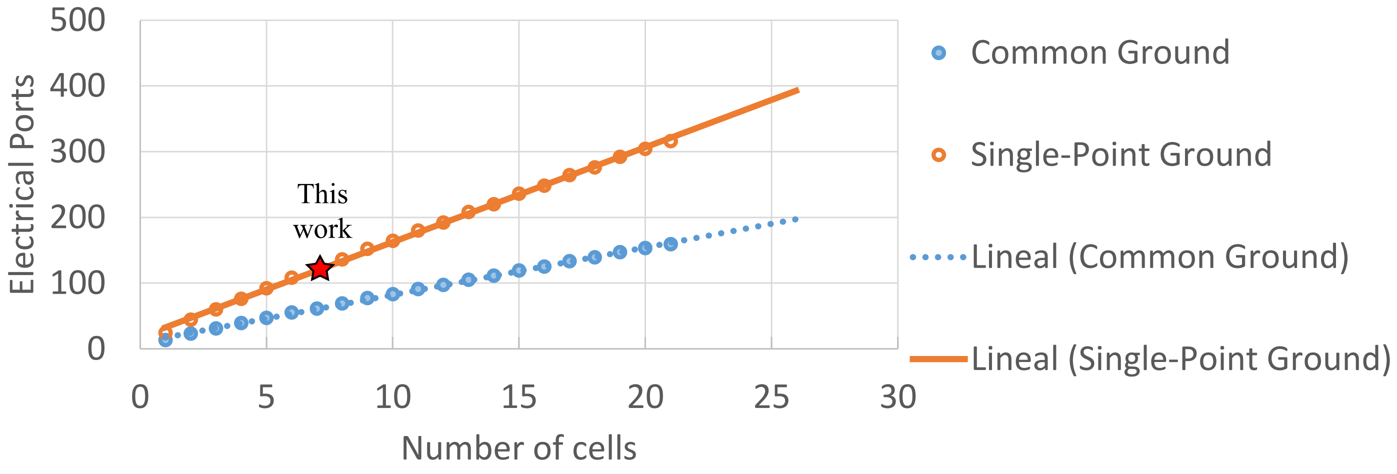

Supplementary Figure 21. Electrical ports versuss number of cells for 2 different ground configurations. **(a)** Single-Point Ground. **(b)** Common Ground.

Supplementary References

[1] Chrostowski, L. & Hochberg, M. Silicon Photonics Design: From Devices to Systems. 1st edn., (Cambridge University Press ,2015).
[2] Reck, M. *et al*. Experimental realization of any discrete unitary operator. *Phys. Rev. Lett.* **73,** 58-61 (1994).
[3] Miller, D. A. B. Self-configuring universal linear optical component. *Photon. Res.* **1**, 1–15 (2013).
[4] Clements, W.R. *et al.* Optimal design for universal multiport interferometers. *Optica* **3**, 1460-1465 (2016)
[5] Madsen, C.K. & Zhao, J. H., Optical Filter Design and Analysis: A Signal Processing Approach. 1st edn.,(Wiley, 1999).
[6] Jinguji, K. Synthesis of coherent two-port lattice-form optical delay-line circuit*. J. Lightw. Technol*. **13,** 73–82 (1995).
[7] Jinguji, K. Synthesis of coherent two-port Optical delay-line circuit with ring waveguides. *J. Lightw. Technol*. **14,** 1882–1898 (1996).
[8] Pérez, D. Gasulla, I., Capmany, J. & Soref, R.A. Reconfigurable lattice mesh designs for programmable photonic processors. *Opt. Express* **24**, 12093-12106 (2016).
[9] Nielsen, M.A. & Chuang, I.L., Quantum computation and quantum information. 1st edn., (Cambridge University Press ,2001).
[10] Carolan, J. *et al.* Universal linear optics. *Science* **349,** 711 (2015).
